# The Mucin Paradox: Decoding the Molecular Strategies of *Akkermansia muciniphila* in Health and Disease

**DOI:** 10.3390/molecules31213945

**Published:** 2026-11-02

**Authors:** Hassan Alarjani, Nawaf S. Alsulami, Hanan A. Alshamrani, Ehab H. Mattar, Alberto Rubio-Casillas, Bassam O. Aljohny, Raed Albiheyri, Faisal Al-Sarraj, Ali Zari, Vladimir N. Uversky, Elrashdy M. Redwan

**Affiliations:** 1 Department of Biological Sciences, Faculty of Science, King Abdulaziz University, Jeddah 21589, Saudi Arabia; halarjani0001@stu.kau.edu.sa (H.A.); nalsulami0381@stu.kau.edu.sa (N.S.A.); halshamrani0087@stu.kau.edu.sa (H.A.A.); emattar@kau.edu.sa (E.H.M.); boaljohny@kau.edu.sa (B.O.A.); ralbiheyri@kau.edu.sa (R.A.); falsaraj@kau.edu.sa (F.A.-S.); azari@kau.edu.sa (A.Z.); 2 Autlan Regional Hospital, Jalisco Health Services, Autlan 48900, Jalisco, Mexico; 3 Centre of Excellence in Bionanoscience, King Abdulaziz University, Jeddah 21589, Saudi Arabia; 4 Department of Molecular Medicine and USF Health Byrd Alzheimer’s Research Institute, Morsani College of Medicine, University of South Florida, Tampa, FL 33612, USA; 5 Therapeutic and Protective Proteins Laboratory, Protein Research Department, Genetic Engineering and Biotechnology Research Institute, City of Scientific Research and Technological Applications (SRTA City), New Borg EL-Arab 21934, Alexandria, Egypt

**Keywords:** *Akkermansia muciniphila*, mucin-degrading enzymes, gut-mucus barrier, host-microbes interaction, next-generation probiotics

## Abstract

*Akkermansia muciniphila* is a next-generation probiotic linked to intestinal barrier integrity and host metabolic well-being. However, the mechanisms underlying its mucin utilization remain incompletely understood. In particular, the temporal hierarchy of enzyme deployment, the subcellular and extracellular-vesicle localization of individual mucin-degrading enzymes (MDEs), the fine substrate specificity of glycoside hydrolases toward distinct O-glycan linkages, and the strain-dependent differences in mucinolytic capacity have yet to be resolved. This review integrates genomic, biochemical, and metabolic data to show how *A. muciniphila* mucin-degrading enzymes (MDEs) degrade mucus, releasing nutrients that support *A. muciniphila* growth and fermentation (acetate and propionate) and cross-feeding by other bacteria, including butyrate producers. It also highlights that *A. muciniphila* exhibits a dual nature: it safely stimulates regulated mucus degradation, improves barrier integrity, and feeds beneficial microbes. In contrast, in specific experimental contexts, such as fiber-deprived or high-sugar murine models, dietary fiber shortage or metabolic stress has been associated with increased mucus degradation, a thinner mucus layer, and greater susceptibility to inflammation and pathogens. The probiotic effects of *A. muciniphila* against metabolic and inflammatory conditions may involve regulated mucin degradation, but benefits may also arise from mechanisms beyond active mucin degradation, including bacterial surface proteins and pasteurized preparations. Unlocking the safe therapeutic potential of this bacterium requires mapping out the spatial, temporal, and strain-specific details of these enzymes. Future research should combine multi-omics and host-interaction studies to clarify enzyme hierarchy, vesicle delivery, and context-dependent impacts.

## 1. Introduction

Mucin-degrading enzymes (MDEs) hydrolyze specific peptide or glycosidic bonds in mucin glycoproteins, thereby disrupting the intact macromolecules and liberating oligo- or monosaccharide fragments, dipeptides, and amino acids [1]. These enzymes, along with other antimicrobial peptides produced by host cells, help regulate pathogenic bacteria, preventing their overgrowth. The corresponding enzymatic activities occur in the gut, thereby affecting the production of short-chain fatty acids (SCFAs) and other metabolites that serve as signaling molecules in host-microbe interactions [2]. Recently, researchers have shown significant interest in microorganisms with evolved enzymatic systems to investigate mucin as a primary nutrient source.

*A. muciniphila* has emerged as a microbe of great interest due to its significant association with host metabolic and gastrointestinal health [3,4,5]. The bacterium has been widely studied as a potential probiotic, often focusing on its protective properties against metabolic syndrome, intestinal dysbiosis, and inflammation [6,7]. *A. muciniphila* is one of the few gut commensals encoding glycoside hydrolases to degrade mucin glycans, such as Mucin 2 (MUC2), the major gel-forming protein of the colon and intestine, creating a protective gel barrier [8,9,10]. MUC2 helps protect the epithelial lining from invaders and maintains the integrity of the mucus layer, playing a significant role in the gut; however, abnormal function has also been associated with conditions such as ulcerative colitis [11]. Surprisingly, colonization by *A. muciniphila* is not only mucin-dependent but also affects mucus homeostasis [12].

It has been found that *A. muciniphila* significantly increases MUC2, the colonic mucin glycoprotein secreted by intestinal Goblet cells [13,14,15]. Accordingly, it has been hypothesized that mucin degradation by MDEs promotes the turnover and renewal of mucin, thereby sustaining the supply of the substrate on which mucin specialists, such as *A. muciniphila*, depend [16]. This paradox of simultaneous mucus degradation and protection is the very embodiment of the MDE-mediated balanced host-microbe interaction.

Within the *Akkermansia* genus, mucin degradation has been well documented; however, elucidating the substrate specificity of *A. muciniphila* strains remains an essential step toward fully understanding this process [17]. Several enzymes enhance growth on complex mucin substrates such as MUC2. However, MDEs differ in substrate specificity and regulatory responses. Thus, knowledge of the diversity and function of these enzymes is central to comprehending the ecological success of *A. muciniphila* within the mucus environment [5,18].

*A. muciniphila* is a Gram-negative, non-sporulating, rod-shaped bacterium that occupies and thrives in the gastrointestinal tract of numerous species, including humans [15]. It is a highly adapted organism to the colon mucus layer, where it participates in cross-feeding and other beneficial symbiotic interactions. This microorganism has been described as important for gut health; high abundance of *A. muciniphila* is correlated with the prevention or alleviation of obesity, type 2 diabetes, and other metabolic diseases [7]. What is still not clear is how exactly mucin degradation activity plays a role in these beneficial responses.

*A. muciniphila* has recently become very popular since it is the only known member of the phylum *Verrucomicrobia* that has a documented probiotic role [15]. It is indeed widely referred to as such. However, the mechanisms by which this organism exerts its beneficial effects are not yet completely understood. Although substantial progress has been made in identifying individual MDEs and their biochemical activities, several critical questions remain unresolved, including: (i) how the expression and activity of these enzymes are regulated in response to dietary, inflammatory, and microbial signals; (ii) whether specific enzymes are preferentially delivered via outer membrane vesicles or remain cell-surface-anchored under defined ecological conditions; (iii) the degree of substrate specificity exhibited by individual glycoside hydrolases and sulfatases toward structurally diverse mucin O-glycans; and (iv) the extent to which strain-level genomic variation modulates mucinolytic efficiency and, consequently, host outcomes.

Recent literature has extensively mapped *A. muciniphila*’s general influence on intestinal barrier function [19] and its broad role in microbiota-mediated disease modulation [4]. Other works have rightly questioned whether its mucinolytic activity is inherently beneficial or detrimental [16], while advanced biochemical studies have detailed the sequential action of carbohydrate-active enzymes on O-glycans [20]. This review distinguishes itself by moving beyond these isolated perspectives to construct a unified molecular framework. Specifically, it elucidates the hierarchical synergy between glycoside hydrolases, specialized proteases, sulfatases, and acetylesterases. Furthermore, it introduces a conceptual distinction between exocrine and endocrine-like mucin-degrading activities. This framework highlights how EV delivery and specific dietary context dictate the transition of *A. muciniphila* from a homeostatic symbiont to an opportunistic pathobiont.

The goal of this review is to analyze the diversity, enzymatic specificity, regulatory mechanisms, and structural organization of *A. muciniphila* glycosidases, proteases, sulfatases, and sialidases as key enzymes involved in mucin degradation. Furhermore, this review analyses the peculiarities of mucin utilization by *A. muciniphila* that not only enables this microorganism to be an active player in the gut microbiota (GM) but also contributes to the maintenance of mucus and intestinal barrier integrity. Exploring the duality of mucus degradation and mucus protection, the review further discusses the role of enzymes in the probiotic activity of *A. muciniphila*.

To ensure methodological transparency, a structured literature selection protocol guided the preparation of this review. Systematic retrieval of sources relied on targeted queries across PubMed, Scopus, and Web of Science databases. Search parameters combined controlled vocabulary and free-text terms, specifically *Akkermansia muciniphila*, mucin-degrading enzymes, glycoside hydrolases, sulfatases, and intestinal barrier integrity. Peer-reviewed primary research and comprehensive reviews published within the last decade were prioritized. Emphasis was placed on studies employing multi-omics approaches, structural biology, and in vivo mechanistic models. Articles lacking empirical data or those failing to address the molecular specifics of host-microbe metabolic crosstalk were excluded.

## 2. *A. muciniphila* as Probiotic

*A. muciniphila* is a member of the human gastrointestinal microbiota that promotes health by supporting mucosal barrier richness and integrity. The bacterium adheres to mucins in mucus layers and degrades these glycoproteins into glycan monosaccharide units, which serve as nutrient sources [21]. This has recently been demonstrated by elucidating the intricate regulatory mechanisms governing the crosstalk between *A. muciniphila*, the host, and the GM [22]. The adhesion of *A. muciniphila* to the intestinal epithelium and its interaction with the host mucosa have been previously investigated in in vitro models [23]. In addition, the ability of *A. muciniphila* to grow with mucin as the sole carbon source has been demonstrated in vitro using gut reactors [24]. Beyond serving as a carbon and nitrogen source for bacterial proliferation, degradation of mucins by *A. muciniphila* generates essential metabolic by-products, namely SCFAs, which are essential for maintaining gut homeostasis and metabolism within the host.

Mucin fermentation by *A. muciniphila* yields mainly acetate and propionate [18,25]. Butyrate is generally produced not by *A. muciniphila* itself but by cross-feeding partners that use mucin-derived sugars or *A. muciniphila* fermentation products (e.g., Anaerostipes caccae in co-culture [26]). Acetate, propionate, and butyrate are physiological metabolites that contribute to intestinal barrier integrity, intestinal homeostasis, and immunity [27,28]. Furthermore, the capacity of *A. muciniphila* to generate SCFAs is intricately connected to its distinctive metabolic features, as the microorganism uses mucin as an energy source and produces beneficial by-products. Lastly, several experimental studies designed to mimic the colonization process have found that mucin supplementation enhances its proliferation and metabolism in response to pH changes [5,6,29]. By being so intimately associated with the mucus layer, *A. muciniphila* occupies an ecological niche unparalleled at the host–microbe interface.

**Figure 1:**
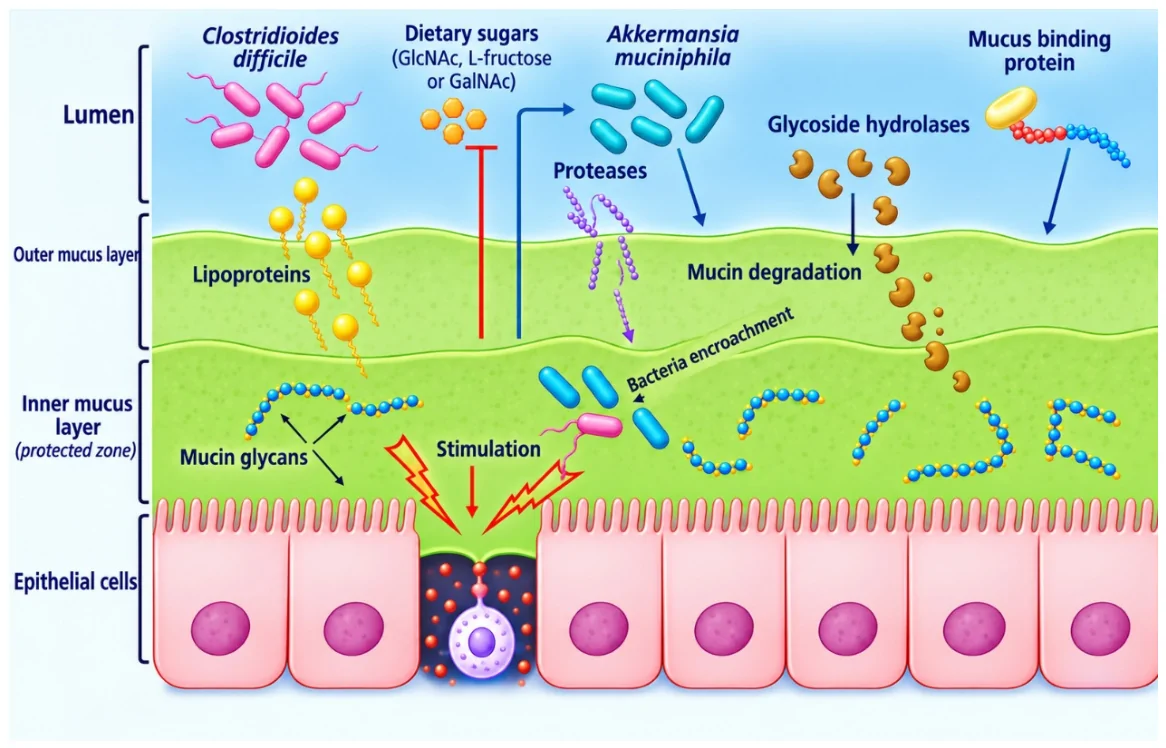
Ecological niche and adhesion of *A. muciniphila* during the mucin degradation process. Two different zones constitute the intestinal mucus barrier: a more permeable outer layer and a compact inner layer that shields the epithelium. This niche is occupied by *A. muciniphila*, which breaks down mucin via glycoside hydrolases and proteases. Mucus-binding proteins facilitate adhesion, and the resultant glycans, such as GalNAc, nourish nearby microorganisms. The spatial arrangement of organisms such as *Clostridium difficile* is influenced by this cross-feeding interaction. As a result, the host epithelium responds specifically to the metabolic byproducts and ecological interactions of these mucus-associated bacteria. GalNAc (N-acetylgalactosamine), GlcNAc (N-acetylglucosamine).

To produce SCFAs that improve the integrity of the epithelial layer while regulating host-microbe interactions, *A. muciniphila* adheres to mucins and uses enzymatic action to break them down into beneficial glycans (Figure 1).

*A. muciniphila* possesses a repertoire of putative bacterial MDEs and/or secreted proteins that impact host–microbe interactions. Current knowledge points to glycoside hydrolases, proteases, sulfatases, and sialidases as the principal enzymes involved in mucin degradation. Biochemical characterization of the purified enzymes from *A. muciniphila* has revealed the presence of 54 glycoside hydrolases and 11 sulfatases that synergistically work to break down the O-glycans in mucin [20]. It has also been found that glycoside hydrolases and sulfatase-encoding genes are upregulated in the presence of mucin, while other putative secreted-protein genes show increased expression when mucin is absent [26]. Similarly, targeted mutagenesis studies have also pointed towards the importance of cell-attached sialidases and fucosidases in the ability of the cell to grow from mucin; their inhibition causes an inability to grow from mucin in vitro [9,20], thereby proving the importance of these enzymes in the degradation of glycosylation [30]. Furthermore, the proteome analysis has also emphasized the importance of multiple β-galactosidases with specific affinities to linkages present in mucin glycans [13,31,32]. The bacterium’s capacity to alter its metabolism in response to available substrates is demonstrated by this differential gene expression.

Gene expression analysis suggests that one digestive strategy involves the stepwise degradation of mucins into low-molecular-weight oligosaccharides, thereby facilitating the release of monosaccharides that are subsequently catabolized [33]. Mucin transcriptomic data show significant upregulation of MDE genes in *A. muciniphila* cultures grown with mucin, confirming substrate-induced expression changes [34]. *In vitro* mucin array experiments support glycan recognition as a prerequisite for degradation by *A. muciniphila*, via specific binding to O-glycan structures on mucins [21]. The sequential breakdown of substances is likely the best way to get nutrients without compromising the renewal of the mucus layer [9,35]. A full-length sialidase has been characterized, and protease homologs may act on mucins and their degradation products [21,26]. All these enzymatic activities emphasize the important role of *A. muciniphila* in keeping mucus homeostasis and enhancing intestinal barrier integrity. Table 1 summarizes key findings from recent studies.

Degradation of mucins by *A. muciniphila* generates essential metabolic by-products, namely SCFAs, which are pivotal for maintaining gut homeostasis. However, this process does not entail exhaustive substrate consumption. Instead, it operates within a controlled turnover cycle where enzymatic cleavage stimulates Goblet cell replenishment. Complete or unregulated degradation would compromise the protective mucus layer; therefore, the bacterium’s beneficial impact relies on a balanced rate of mucinolysis that supports, rather than depletes, the inner mucus barrier.

## 3. *A. muciniphila* as an Important Probiotic Producing MDEs

*A. muciniphila* is a promising new probiotic currently undergoing extensive preclinical and clinical research. It stands out from better-established probiotic candidates because it thrives in the gut mucus layer and utilizes mucin glycans as nutrients, producing mucin-degrading enzymes in its natural habitat [34]. Recent genetic mutagenesis analyses have identified particular loci critical to mucin degradation and stable mucinophage colonization in a laboratory setting, thereby establishing the relationship between mucin degradation and its ecological fitness in the mucus habitat [9]. These results clearly illustrate the notion that mucin degradation is not a random event but rather an innate and critical ability of *A. muciniphila*.

Advanced molecular techniques and metabolomics have clarified how this bacterium adapts to the high-mucin environment of the intestine. A recent study of heterologous expression and biochemical analysis of a novel α-galactosidase demonstrated unexpected activity towards mucin-derived glycans, demonstrating the continued discovery of novel enzymes for mucin degradation in this species [41]. Simultaneous metabolomics and lipidomics of *A. muciniphila* grown under conditions of high mucin availability reflect dramatic changes in carbohydrate metabolic pathways, cell membrane lipid content, and energy-related metabolism, suggesting synchronized metabolic responses to higher mucin availability [39]. Collectively, these laboratory findings c confirm again that mucin degradation is coupled with systemic metabolism.

Based on the available knowledge, the beneficial effects of *A. muciniphila* in conditions such as obesity and inflammatory bowel disease can be attributed to a combination of mucin degradation and the ability to utilize mucin-derived glycans. *In vitro* synthesized mucosal communities show that while *A. muciniphila* degrades mucin, they remain functionally compatible with other mucus-associated microorganisms, underscoring their medically important role [30,39]. Further studies using human colonoid tissue cultures show mucin-mediated gene induction related to uptake, adherence, and pili biogenesis in a human transcriptome [40]. These studies establish that degradation of mucin by *A. muciniphila* is a regulated process to maintain host-microbe homeostasis.

Although mucin removal from the growth medium markedly altered the transcriptome, proteome, and metabolome, it increased the capacity for carbohydrate degradation and resistance to bile acids. *A. muciniphila* cultured under high-mucin conditions retained significant probiotic properties [34]. Likewise, its capacity to degrade mucin does not appear to compromise its suitability as a great probiotic [39]. Thus, the MDEs of *A. muciniphila* are hypothesized to facilitate cycles of mucin degradation and replenishment without undermining the overall integrity of the intestinal mucus barrier.

Considering the well-documented contribution of this bacterium in ensuring a healthy status of the host cell, its boundary conditions, such as host fiber deprivation and extreme metabolic stress, are not yet fully elucidated, and future research must ensure that it recalibrates its MDE expression in scenarios where nutrients are scarce. This will be crucial for ensuring the integrity of *A. muciniphila*, particularly within a multi-kingdom ecosystem, where competitive ecological pressures may influence its probiotic activity.

## 4. Types and Categories of MDEs

MDEs are enzymes that degrade mucin glycans into oligosaccharides or other small molecules. The mucin-degrading capability is demonstrated by a variety of species [42]. In the human gut, *A. muciniphila* was the first identified bacterium to exhibit mucin-degrading activity [25], indicating its presence within the mucus layer. Intestinal colonization by *A. muciniphila* allows the bacterium to use mucins not only as a carbon source but also as a nitrogen source to fulfill other nutritional and growth requirements [24,43].

The abundance of *A. muciniphila* in infants and healthy adults may reach approximately 1–3% of the total bacterial population [25], a finding which underscores the medical significance of this bacterium for gut homeostasis. Experimental evidence shows that this bacterium can degrade mucin, producing monosaccharides, oligosaccharides, and/or amino acids during mucin metabolism [44]. These properties indirectly influence other gut bacteria, thereby releasing degradation products of glycoproteins and ultimately controlling inflammation. The exact *A. muciniphila*–mucin connection remains poorly understood from a functional standpoint, necessitating further studies to fully elucidate the enzymatic activity harbored by this bacterium [20]. Notably, experiments with purified *A. muciniphila* proteins have demonstrated the enzymatic activity of some of these proteins over mucin [41]. In addition to determining mucin-degrading ability, it is imperative to understand the functional characteristics of the MDEs secreted by *A. muciniphila*.

Recent biochemical, proteomic, and structural biology studies using recombinant enzymes, targeted gene deletions, and growth on defined mucin glycans [38], and mass spectrometry-based glycan analysis have begun to reveal the exact enzymatic process involved in mucin degradation by *A. muciniphila* [20]. These studies indicate that mucinolysis relies on a coordinated enzyme repertoire whose members act on both the glycan and protein backbones of mucin molecules. Coordinated or synergistic activity alone does not provide evidence for the assembly into a physical enzyme complex, and no such complex has been shown.

### 4.1. Exocrine-like and Endocrine-like Functions of A. muciniphila MDEs: A Proposed Conceptual Framework

In this review, the terms “exocrine-like” and “endocrine-like” are introduced as a part of a proposed conceptual framework, not as an established biochemical or enzymological classification. Only the first category comprises mucin-degrading enzymes in the strict sense, namely enzymes that directly hydrolyze mucin peptide or glycosidic bonds, or remove sulfate and O-acetyl substituents from mucin glycans. The second category does not describe a separate class of enzymes. It refers to the downstream, metabolite-mediated host signaling (for example, by short-chain fatty acids, SCFAs) that follows microbial fermentation of mucin-derived products. The terminology does not imply that the bacterium secretes hormones.

#### 4.1.1. Exocrine-like (Direct Mucin-Degrading) Activities (Exo-MDEs)

Exocrine-like mucin-degrading enzymes (Exo-MDEs) act directly on mucin glycoproteins in the mucus layer [45]. These MDEs are microbial proteins that are cell-surface-associated or secreted and that target specific bonds within mucin O- and N-glycans [21], facilitating mucin degradation into smaller oligosaccharides and ultimately into monosaccharides [18,20]. Some Exo-MDEs belong to the sialidases, fucosidases, β-galactosidases, hexosaminidases, and sulfatases known as carbohydrate-active enzymes (CAZ enzymes), which target terminal structures and inner mucin bonds [21,26]. In *A. muciniphila*, the key enzymes include glycoside hydrolases, sialidases (GH33), and fucosidases (GH29 and GH95) that target bacterial cell surfaces. They play vital roles in bacterial growth when mucin is used as the substrate [26].

For instance, ten glycoside hydrolases in *A. muciniphila* were found to remove GHs sialyl and fucosyl caps from mucin O-glycans, triggering the breakdown of mucin and allowing them to access the sugar chains beneath [26]. The activity of these enzymes is characterized by cell surface binding to mucin and by enabling nutrient sharing with other bacteria [21,26]. Another example of Exo-MDEs is the identification and biochemistry of the secreted β-galactosidases (Amuc_0771, Amuc_0824, and Amuc_1666) of *A. muciniphila*, which recognize specific galactosidic bonds abundant within mucin structures [32]. These enzymes specifically degrade the glycan moieties of porcine mucin, thus verifying their ability to catalyze mucin degradation outside cells [30,32].

**Figure 2:**
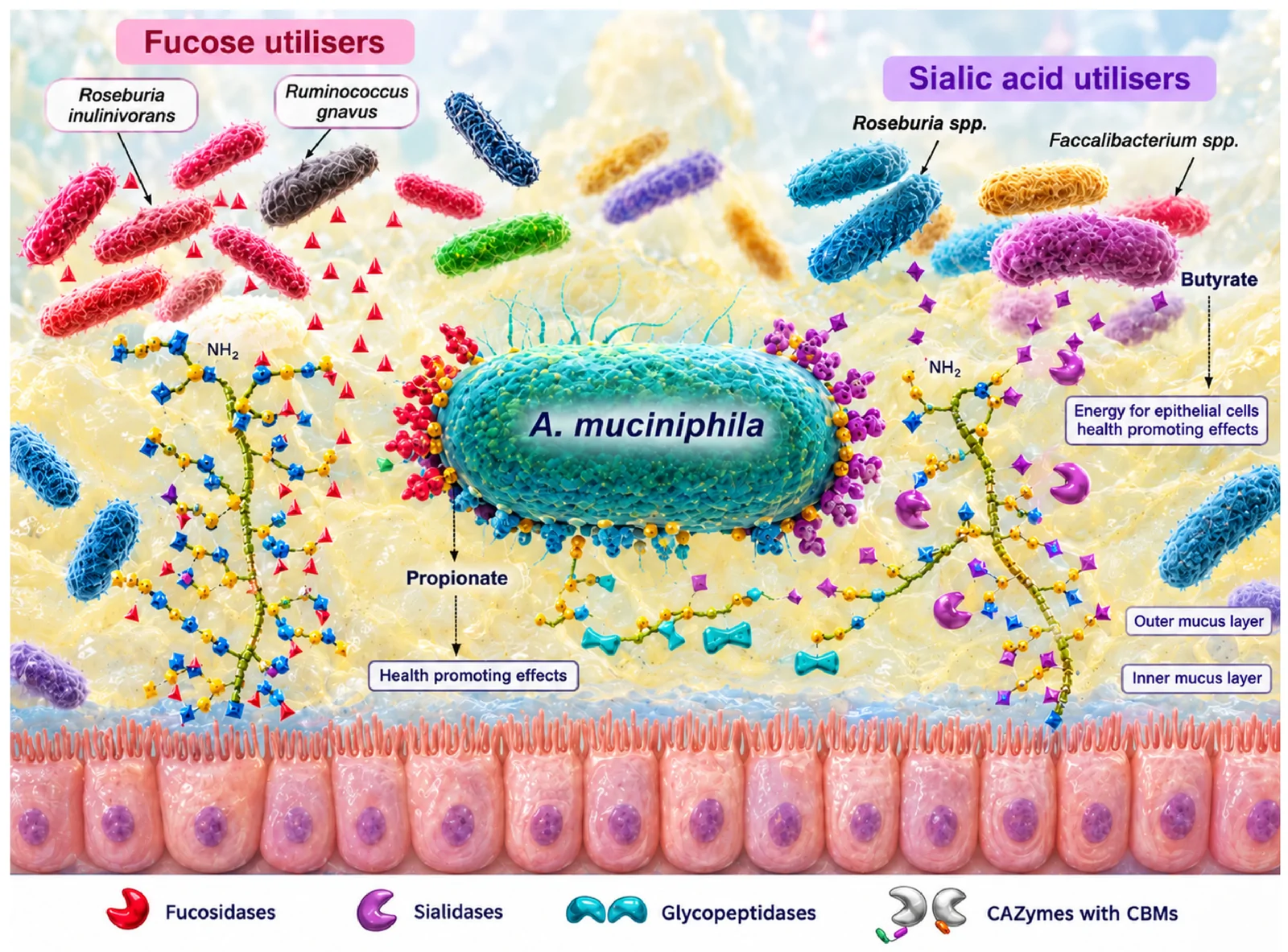
Conceptual model of mucin degradation and microbial cross-feeding by *A. muciniphila* (adapted from data presented in ref. [26]). Propionate is produced by *A. muciniphila*; butyrate is produced by cross-feeding partners (*Roseburia* spp., *Faecalibacterium* spp.). CBM, carbohydrate-binding module; CAZymes, carbohydrate-active enzymes.

Cataloguing the CAZymes encoded in the *A. muciniphila* genome has provided an essential foundation; future studies should now define how these enzymes are regulated and ordered, that is, the hierarchy of activities used by Exo-MDEs. We know, for example, that certain glycoside hydrolases, such as GH33 and GH29, have been identified; however, the spatiotemporal logic governing how these enzymes sequentially cleave caps from a complex O-glycan remains incompletely understood. Exploring how these enzymes bound to or secreted onto a biofilm, function and compete in a polymicrobial context is essential for understanding nutrient economies. And finally, determining how a host or dietary factor influence these exocrine tools is also needed. In summary, the mechanisms involved in mucin degradation are schematically depicted in Figure 2 below.

#### 4.1.2. Endocrine-like (Downstream Metabolite-Mediated) Effects

In contrast to the direct enzymatic activities described in Section 4.1.1, the second tier of this conceptual framework addresses the downstream physiological consequences of mucinolysis. The endocrine-like category is not defined by direct mucin cleavage and does not constitute a distinct class of enzymes. It denotes the host signaling that follows mucin degradation. Once Exo-MDEs release mucin-derived sugars, these are fermented by *A. muciniphila* and by cross-feeding partners, and the resulting metabolites (SCFAs and others) act on host receptors and pathways [45,46]. This metabolite-mediated signaling should be distinguished from (i) direct mucin degradation and (ii) signaling by *A. muciniphila* proteins that are not mucin-degrading enzymes, such as the outer-membrane protein Amuc_1100 and the secreted GLP-1-inducing protein P9 [46]. In fact, once mucin is degraded by exocrine-like enzymes, the liberated monosaccharides and SCFAs modulate host pathways, including immune responses, barrier integrity, and metabolic networks [45]. For instance, mucin degradation products can be metabolized into SCFAs, such as acetate, propionate, and butyrate, by *A. muciniphila* itself (acetate and propionate) and by cross-feeding commensal microbes (butyrate) [47]. These SCFAs influence host gene expression and immunomodulatory pathways via G protein-coupled receptors and histone deacetylase inhibition, affecting epithelial health and systemic metabolism [36,48,49].

**Figure 3:**
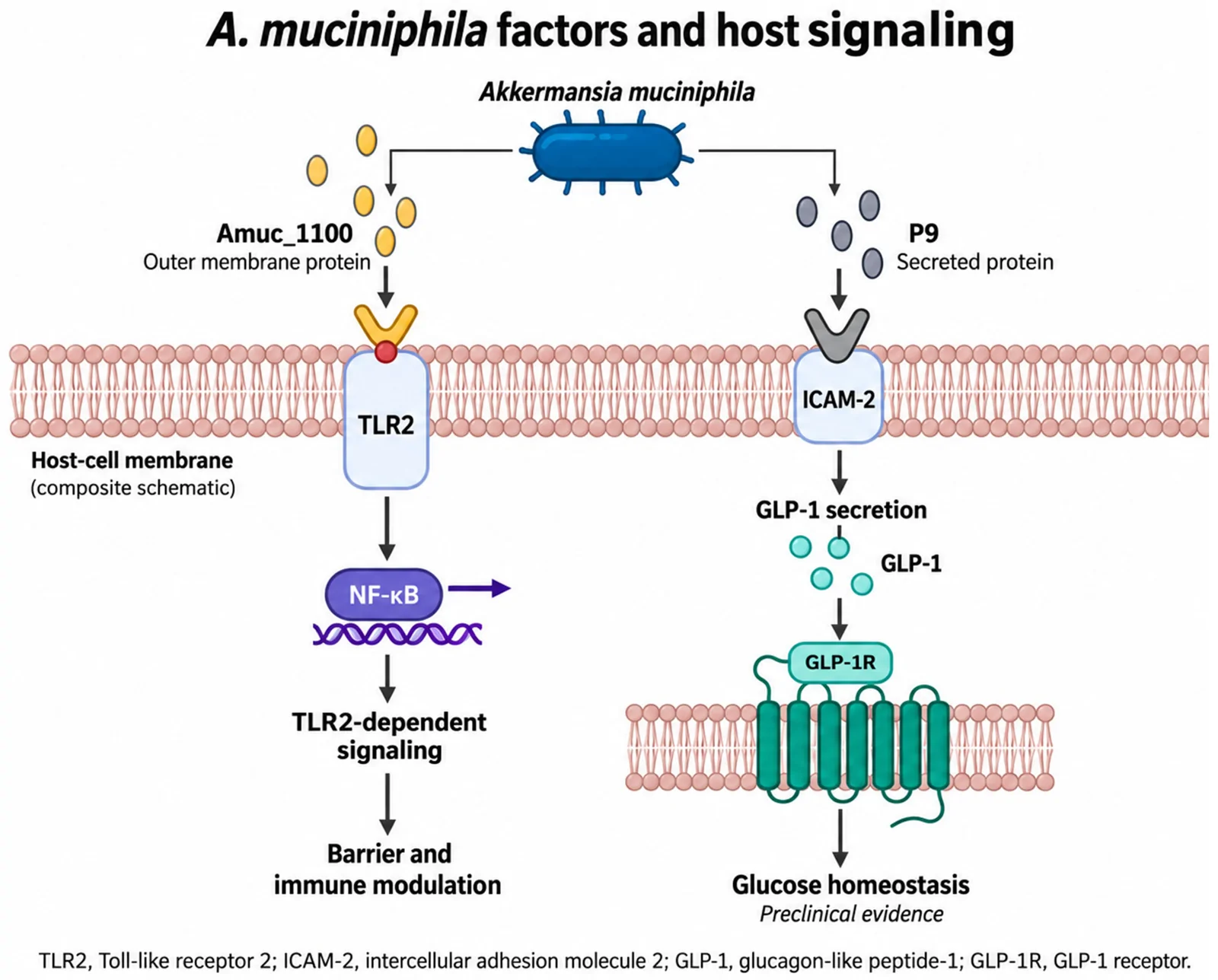
Selected *Akkermansia muciniphila*-derived proteins and their participation in host signaling. The outer membrane protein Amuc_1100 activates Toll-like receptor 2 (TLR2) and downstream signaling by nuclear factor κB (NF-κB). This scheme illustrates immune responses and epithelial barrier function and does not imply that NF-κB activation always suppresses inflammation or explains barrier function directly. The secreted protein P9 interacts with intercellular adhesion molecule 2 (ICAM-2) and stimulates glucagon-like peptide-1 (GLP-1) secretion in experimental models. The lower separate membrane shows subsequent GLP-1 receptor (GLP-1R) signaling in a target cell. Colored protein symbols distinguish Amuc_1100, P9, and GLP-1; arrows show the direction of the interactions summarized. The upper membrane is a schematic illustration and does not imply the occurrence of all events in the same cell. The metabolic effects illustrated are derived primarily from preclinical studies. Receptor shapes and molecular dimensions are illustrative.

Although *A. muciniphila* does not produce hormones classically defined as endocrine factors, microbial metabolic pathways generate signaling metabolites that affect host tissues beyond the mucus niche; within the proposed framework, these systemic effects are termed endocrine-like [50]. For example, SCFAs produced from mucin degradation circulate and bind host receptors, contributing to mucin secretion, immune modulation, and metabolic regulation. In summary, Exo-MDEs mediate the primary breakdown of mucin polymers in the mucus layer, whereas the endocrine-like effects described here are attributable to downstream metabolites acting as messengers in host–microbe crosstalk, not to a separate enzyme class. Signaling pathways by the non-enzymatic *A. muciniphila* proteins Amuc_1100 and P9 are summarized in Figure 3.

However, the future of the Endo-MDE framework should involve bridging the gap between microbial catabolism and systemic host signaling. Studies need to transcend descriptive taxonomies toward a mechanistic mapping of how specific enzymatic repertoires dictate metabolite fingerprints. By using systems biology to integrate multi-omic data, we can decode the synergistic effects of mucin-derived metabolic soups on host receptors. Quantitative thresholds for these interactions will enable precision therapeutics, in which engineered commensals modulate the endocrine system. Table 2 below summarizes the principal mucin-degrading enzymes of *A. muciniphila*, distinguishing activities demonstrated experimentally from functions that have been predicted or remain only partly characterized. Substrate specificity is stated only where it has been shown.

### 4.2. MDEs in Extracellular Vesicles

Mucins serve as a first-line defense mechanism, being secreted to reinforce the gastrointestinal barrier against pathogens, toxins, and other harmful agents, thereby forming a protective mucus layer over the intestinal epithelium [42]. *A. muciniphila*, which is considered a next-generation probiotic candidate, belongs to the *Verrucomicrobia* phylum and is a mucin-degrading bacterium that can grow on mucin under laboratory conditions as a sole source of carbon and nitrogen [9]. This capacity for mucinolysis is thought to play an important role in the beneficial effects attributed to the bacterium in maintaining host metabolic and immune homeostasis, as has been extensively described in the literature [42].

**Figure 4:**
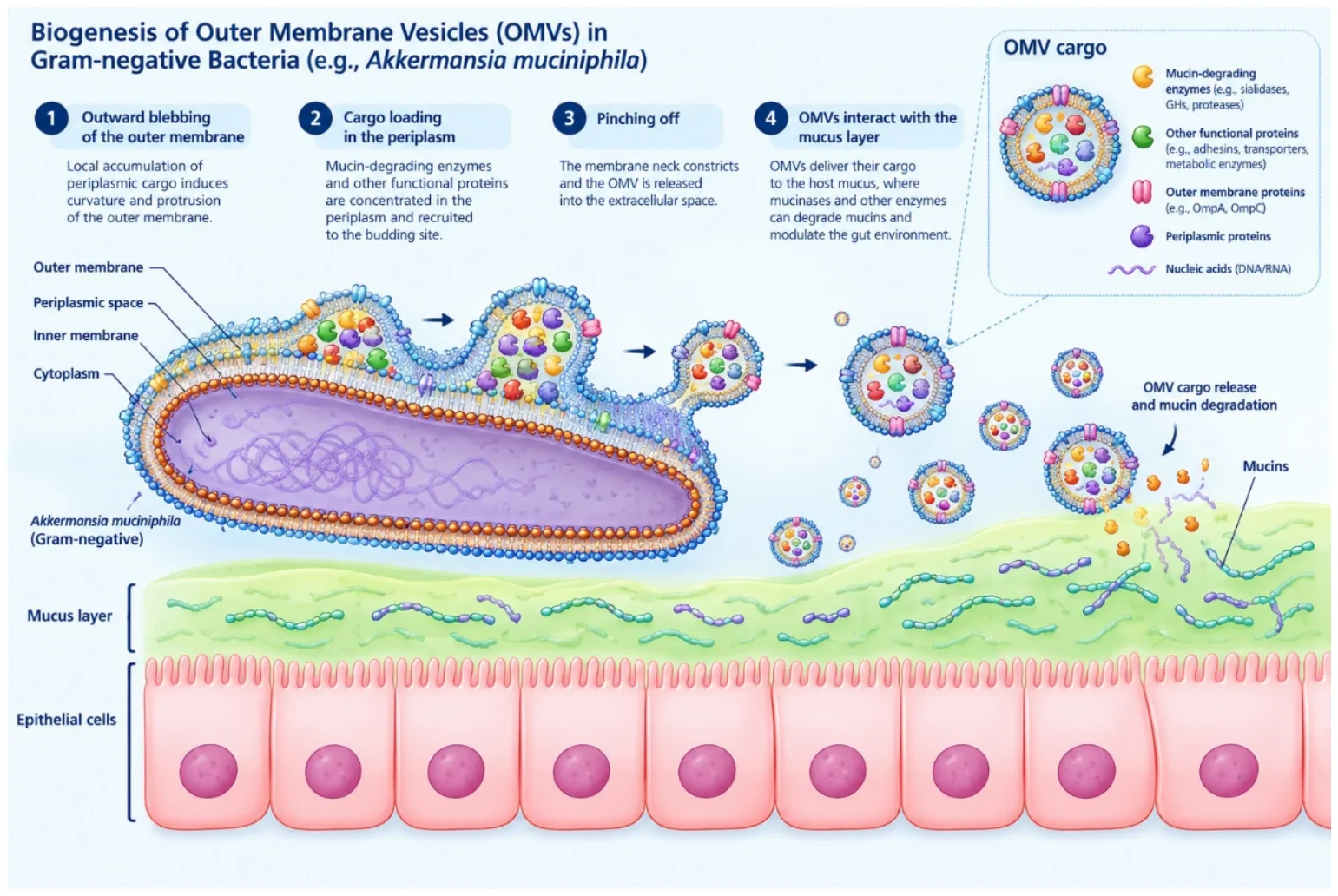
Biogenesis and structural representation of OMVs in *A. muciniphila*. The diagram illustrates the outward blebbing of the Gram-negative outer membrane, driven by localized accumulation of periplasmic content and lipid microdomains. This process results in the fission of OMVs from the membrane. OMVs encapsulate MDEs, nucleic acids, and lipopolysaccharides for targeted delivery into the host intestinal mucus layer. Arrows show the direction of the interactions summarized.

*Akkermansia muciniphila*-derived Extracellular vesicles (EVs) have been characterized in previous studies. The protein profile of the organism has also been studied to ascertain the EV-associated proteins [32,51]. These data justify the study of vesicles as mediators of bacterial–host interactions. Nevertheless, the presence of a protein in an EV preparation cannot be regarded proof of its location in the vesicle, enzymatic activity, or delivery to a physiological substrate. Transport of the enzymes by vesicles could potentially extend mucinolytic activity outside the bacterial surface, but this hypothesis needs experimental verification of enzyme localization, retention of catalytic activity, and substrate degradation by vesicles. Figure 4 illustrates the bacterial vesicle model and the proposed enzyme-delivery function.

Complete genomic analysis indicates that *A. muciniphila* possesses an enormous genome encoding MDEs, including glycoside hydrolases, proteases, sulfatases, and sialidases, which incrementally degrade mucins into peptides and ultimately amino acids that serve as nutrients for bacteria [51,52]. Demonstrated vesicle cargo [51] should therefore be distinguished from the proposed packaging and delivery of MDEs described above. Gram-negative bacteria, including *A. muciniphila*, release outer-membrane vesicles (OMVs). Whether *A. muciniphila* MDEs are selectively packaged into these vesicles has not been established; it has been proposed that bacterial MDEs may be vesicle-associated [10,25]. If confirmed, this would provide a potential mechanism for delivering enzymes beyond the immediate bacterial cell surface, thereby facilitating localized mucin remodeling within the mucus layer [53]. The demonstrated vesicle cargo [51] should therefore be distinguished from this proposed packaging and delivery of MDEs.

Characterizing the possible packaging of MDEs into EVs is critical to understanding how *A. muciniphila* plays its ecological role in turning over mucins with a broader impact on microbiome–host interactions. Such enzymatic activities might be involved in supporting balanced cycles between mucin degradation and renewal, while promoting cross-feeding among microbes and maintaining the integrity of the intestinal barrier system.

### 4.3. Characteristics of MDEs

In the process of breaking down mucins, *A. muciniphila* releases other metabolites known as glycans, which are usually used by other microbes [30]. This process increases the efficiency of metabolic cross-feeding among different microbial pathways, while promoting the establishment of stable microbiomes [30]. Genomic studies also validate this process of functional specialization, as *A. muciniphila* possesses a relatively large genome with several transporters associated with mucus-related genes [52], which affirms its adaptation at the mucosal site [20]. These highlight the prime importance of MDEs for the metabolic adaptation of *A. muciniphila*.

*A. muciniphila* is also a strict anaerobe and a dominant gut symbiont in humans, and it has adopted a specialized metabolic pathway to utilize mucin components as its energy source [25]. The enzymes responsible for mucin degradation are also involved in bacterial catabolic processes, acting in a coordinated and, on the substrates examined, largely sequential manner, cleaving O-glycans, then saccharides, and peptidic bonds to obtain monosaccharides, oligosaccharides, and amino acids as energy sources for the bacterium [20]. This degradation requires several coordinated steps and encompasses a coordinated microbial strategy for the efficient breakdown of mucin components [20]. Mucin comprises a large sugar-protein complex produced by specialized epithelial cells known as goblet cells, forming a protective barrier between the intestinal epithelium and its lumen [33].

Finally, it has been found that the structure of mucins has a protein backbone heavily modified with O-linked carbohydrate chains [54]. These chains can be an important source of carbon and energy for specific bacteria that live in the gut [25]. *A. muciniphila* has genes for many distinct types of enzymes that can degrade mucin [55]. These include glycoside hydrolases, sulfatases, sialidases, proteases, and polysaccharide lyases [18]. Together, these enzymes enable the step-by-step breakdown of complex structures found in mucins [56].

## 5. Mechanisms of MDE Action

The ability of *A. muciniphila* to colonize the mucosal layer is based on a repertoire of mucin-degrading enzymes, which can be grouped into five functional categories according to the reaction catalyzed or the substrate modification targeted: glycoside hydrolases (GHs), proteases, sialidases, sulfatases, and acetylesterases [18,25]. These categories are not mutually exclusive enzyme classes. Sialidases (GH33) are themselves glycoside hydrolases but are classified separately because they remove a distinct terminal residue that gates access to the underlying glycan. An in-depth critical analysis of these enzymes reveals a highly coordinated, sequential mechanism of action that is critical for nutrient acquisition and gut barrier modulation [2,57]. The basic architecture of mucin, consisting of a protein backbone with a high degree of O-glycosylation and complex, often branched glycan structures, underscores the need for such a multi-enzymatic degradation process [54].

### 5.1. Glycoside Hydrolases

Members of this category are commonly designated as backbone degraders. They are the primary enzymes that cleave these complex O-glycan chains, which account for most of the mucin molecules. Rather than being random in their activity, the glycoside hydrolases (GHs) of *A. muciniphila* have exquisite specificity. For example, the GH33 family of enzymes comprises sialidases, as discussed above. The GH20 and GH84 enzyme families are involved in the cleavage of core chains. Recent studies have shown that some of the GHs of *A. muciniphila* can cleave the core 3 GlcNAcβ1-3GalNAc and the core 6 GlcNAcβ1-6GalNAc O-glycans, primarily found in intestinal mucins such as MUC2 [13]. Their mode of action also includes an endo-acting mechanism that cleaves glycosidic bonds within the glycan chains, resulting in the release of smaller oligosaccharides. This is different from the mode of action of exo-glycosidases, where they nibble the glycan chains from the non-reducing ends. This dual GHs activity appears to be a disassembly line, in which the endo-cleavage activity creates a variety of ends for the exo-glycosidases to act on, thus maximizing energy extraction from the glycan source [2].

### 5.2. Sialidases

Sialic acids, especially the O-acetylated types, which are present in large quantities, can also form the terminal ends of mucin glycan structures. They can serve as a physical barrier against bacterial breach, while also providing immune evasion mechanisms through molecular mimicry. However, *A. muciniphila* uses exo-acting sialidases, which are glycoside hydrolases that predominantly belong to the GH33 family. These enzymes can specifically degrade terminal sialic acids by unmasking the glycans. The most important aspect of these enzymes is that they are not all the same. Some of them can degrade α2-3-linked sialic acids, while others can degrade α2-6-linked sialic acids. This is because the sialylation of the mucin glycan structures can vary [2]. By removing the sialic acids, these enzymes can perform a gatekeeping role. The underlying glycan structures thereby become accessible to the action of other GHs. Enzymatic removal of terminal sialic acid should be distinguished from consumption of the released sugar. In the study of Shuoker et al. [28], the released sialic acid did not contribute to the growth of *A. muciniphila* under the conditions tested; instead, it supported metabolic interactions (cross-feeding) with other gut bacteria. Sialidase activity itself is, however, important for growth on mucin because it exposes the underlying glycan structures [28].

### 5.3. Sulfatases

Sulfatases act on sulfate ester substitutions that are part of the mucin glycan substrates; the sulfate groups belong to the glycans, not to the enzymes. Sulfate is found on residues such as GlcNAc and GalNAc of mucin O-glycans, where the negative charge is thought to increase mucin viscosity and resistance to microbial degradation. Sulfatases hydrolyze these sulfate ester bonds, releasing inorganic sulfate and removing the negative charge. *A. muciniphila* harbors sulfatases that hydrolyze these sulfate substituents. They are described as acting in an “exo-sulfatase” mode, removing sulfate groups from the non-reducing end of the glycan [20,25]. This removal of negatively charged groups is not just an action but a prerequisite. Many GHs cannot accommodate sulfated groups in their active sites. Therefore, the removal of sulfate substitutions from the glycan substrate can expose the glycan to subsequent GH action. This is an important aspect of the hierarchical degradation of mucin.

### 5.4. Proteases

Although GHs are responsible for glycan degradation, proteases are the primary enzymes that break down the protein backbone of MUC2, which serves as a scaffold, attaching these glycan bristles. *A. muciniphila* utilizes several types of proteases with different specificity profiles. In fact, it is the first protease to cleave the N-terminal domain of MUC2, which is required for disulfide bond-mediated polymerization [37]. This initial cleavage event is crucial for MUC2 degradation, as it enables the breakdown of large net-like MUC2 multimers into smaller soluble monomers. Other proteases, most likely serine or cysteine proteases, subsequently degrade MUC2 monomers into even smaller peptides and eventually, amino acids. These specific proteases cleave after aromatic amino acids (Phe and Tyr) or polar amino acids [18], thereby fully degrading the MUC2 protein backbone down into small peptides and free amino acids. Thus, members of this group act by disassembling the scaffolds from the glycans in an elegant two-step process.

### 5.5. Acetylesterases

The final class of enzymes is the acetylesterases. They act on the O-acetyl groups of sialic acid, which are referred to as O-acetylesterases, as well as other carbohydrates such as GalNAc. The mechanism of action of this enzyme class is the hydrolysis of the bond that holds the acetyl group to the carbohydrate, thereby fine-tuning the substrate. The biological importance of this enzyme class is two-fold. As with sulfation, O-acetyl substitution of the glycan can hinder GH activity. Removal of the relevant substrate O-acetyl groups by this enzyme class is therefore required for GHs to act on the affected carbohydrate chains [18,25].

**Figure 5:**
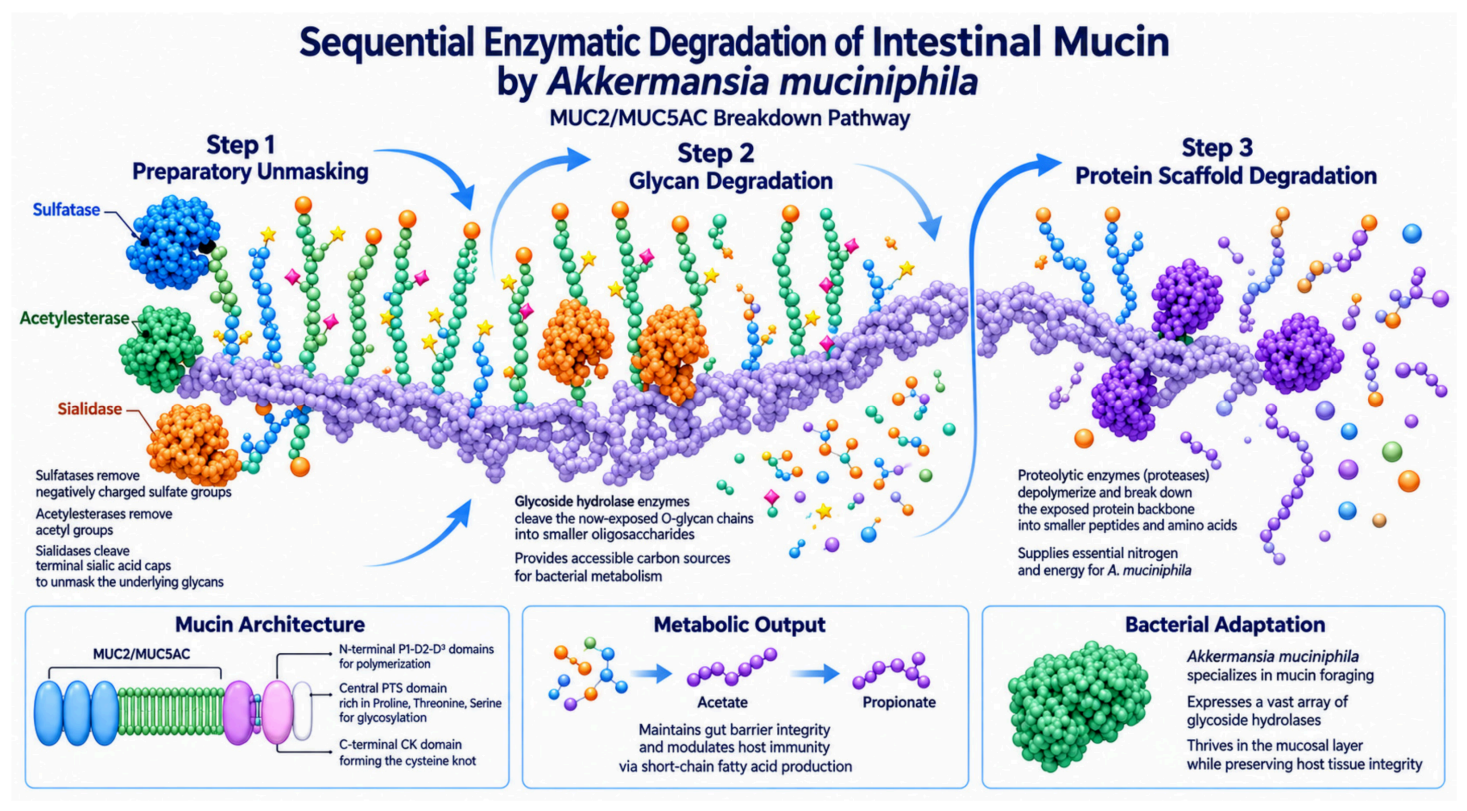
Sequential enzymatic degradation of intestinal mucin (MUC2/MUC5AC) by *A. muciniphila*. The diagram illustrates the hierarchical breakdown of the mucin polymer.

The presence of five functional categories is likely not coincidental and is consistent with a coordinated, multi-step approach (see Figure 5). In a commonly proposed model, sulfatases and acetylesterases remove sulfate and O-acetyl substitutions, and sialidases remove terminal sialic acid caps, which in turn allows glycoside hydrolases to cleave the exposed glycan chains. The order of these activities is likely to depend on the substrate structure, and a universal degradation sequence has not been demonstrated. At the same time or subsequently, the proteolytic enzymes depolymerize and degrade the newly exposed or degraded protein scaffold. This multi-step synergistic approach is directed toward the “promiscuous” substrates of MUC2 and MUC5AC [35,56]. Bakshani et al. [20] showed that this enzyme repertoire degrades to completion the specific O-glycans examined experimentally. This should not be taken to mean complete degradation of the intact, high-molecular-weight mucin polymer, which has not been demonstrated.

## 6. Balancing Between the Advantages and Disadvantages of *A. muciniphila* as a Probiotic

*A. muciniphila* is epitomized as a promising probiotic. Mucins, which function as protective barriers for epithelial cells and are critical for host–microbe crosstalk, are one of its few carbon sources [22]. Biochemical investigations have shown that this gut commensal secretes mucin-degrading enzymes that could affect host microbiota and health [54,58]. These mucolytic capabilities are assumed to enhance the commensal’s own proliferation, owing to the glycan content. Still, they may also produce mucin oligosaccharides that reach monomeric forms, thereby influencing epithelial cell communication [54,58]. Since mucins are also critical for gut health, elucidating the trade-off between mucin degradation and subsequent beneficial or detrimental effects to the host would be relevant [22,37]. Recent reviews and mechanistic investigations highlight their positive role in metabolic disorders, mucosal barrier maintenance, and immune modulation. Below is a synthesis of key findings, with references to the underlying literature.

In an exploratory proof-of-concept randomized, placebo-controlled study of overweight and obese volunteers with insulin resistance, three months of *A. muciniphila* supplementation was safe and well tolerated, and the pasteurized preparation improved insulin sensitivity and reduced insulinemia and plasma total cholesterol; fasting glycemia was not significantly reduced [59]. The report by Santacruz et al. is an observational study in pregnant women that associated gut microbiota composition with body weight and biochemical parameters; it did not test supplementation and cannot support supplementation efficacy [60]. In diet-induced obese mice, live or pasteurized *A. muciniphila* and the outer-membrane protein Amuc_1100 improved metabolic parameters and reduced adiposity and inflammatory markers [6,58]. In animal models, research has also shown that when administered at appropriate doses, *A. muciniphila* enhances tight-junction protein expression, increases mucus thickness, and decreases the synthesis of pro-inflammatory cytokines, such as tumor necrosis factor alpha and interleukin 6 (IL-6) in murine colitis and “leaky gut” models [61].

It is now known that GM dysbiosis is implicated in the development of systemic lupus erythematosus (SLE) in humans [62,63]. In this regard, a recent study demonstrated that administration of *A. muciniphila* decreased inflammatory markers in mouse models of systemic lupus erythematosus (SLE), specifically reducing levels of IL-6 and IL-17 and increasing levels of IL-10, an anti-inflammatory cytokine [64]. In addition, in mouse models of immunosuppression, catabolic syndrome, and persistent inflammation, *A. muciniphila* modified the GM. Beyond increasing levels of *Muribaculaceae*, *Lachnospiraceae*, *Lachnoclostridium*, and *Parabacteroides goldsteinni*, both live and pasteurized *A. muciniphila* reduced the abundance of bacteria associated with illness and inflammation [65]. Another investigation found that this bacterium reduces intestinal inflammation by stimulating the generation of regulatory T-cell populations [66]. In cancer immunotherapy, higher intestinal abundance of *A. muciniphila* has been associated with clinical response to PD-1 blockade in patients (observational findings) [67,68], and, in mouse experiments, oral supplementation with *A. muciniphila* restored the efficacy of PD-1 blockade [68]. These data indicate association and preclinical immunomodulatory potential; they do not establish a clinical benefit of supplementation. In other studies, supplementation with *A. muciniphila* reduced depressive-like behavior in an animal model by modulating gut-brain axis signaling and serotonin pathways [69,70]. Associations and preclinical findings have also been reported for neurological conditions, including epilepsy [71], Parkinson’s disease [72,73], and autism spectrum disorder [74]; a causal therapeutic benefit of supplementation remains to be established. For Alzheimer’s disease, the cited work [75] examined ketogenic and low-fat dietary interventions in adults at risk for Alzheimer’s disease and does not test bacterial supplementation; it therefore does not support symptom alleviation by *A. muciniphila*.

**Figure 6:**
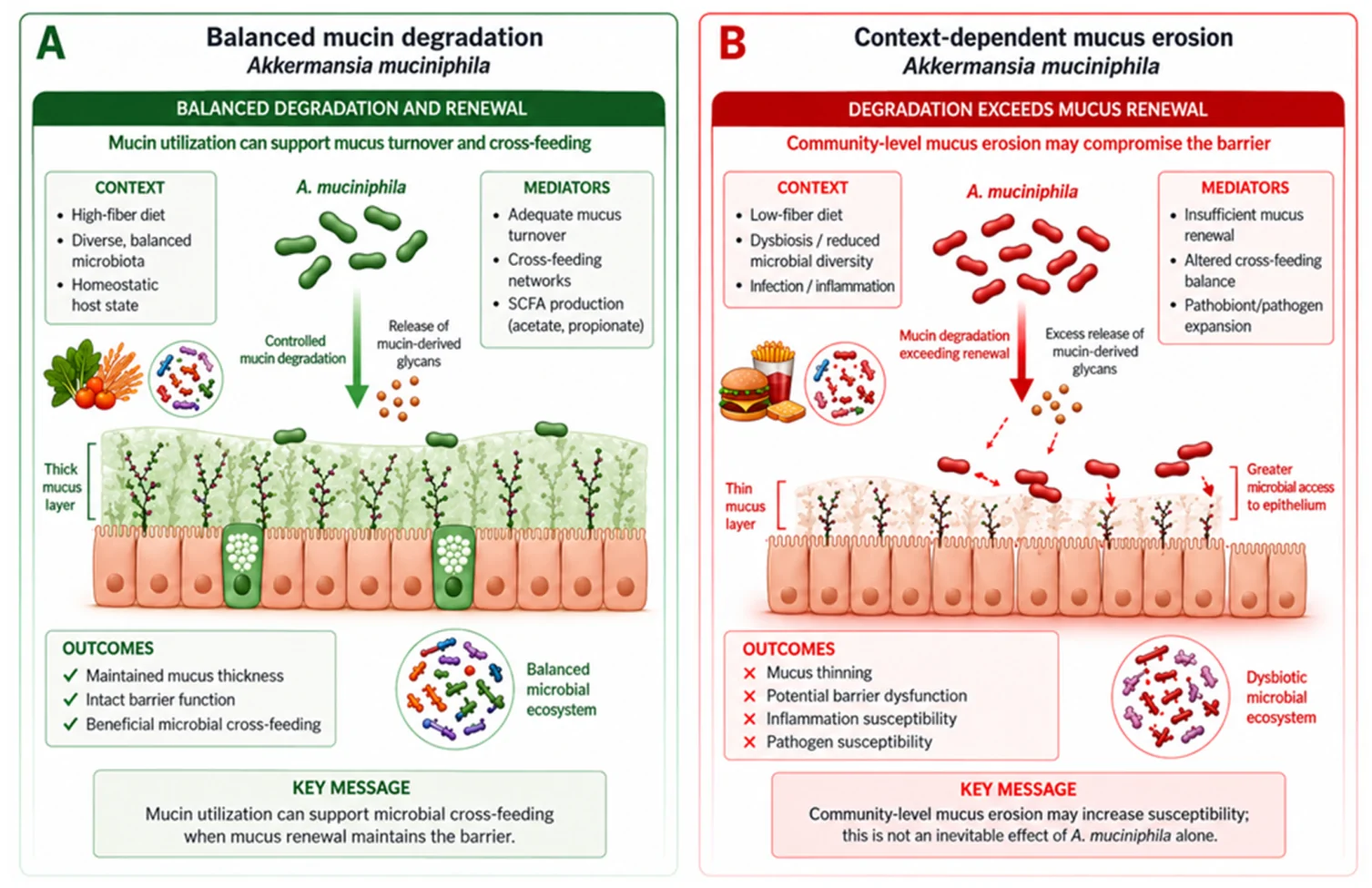
Conceptual model illustrating the dual role of *A. muciniphila*-mediated mucin degradation. (**A**) If the balance between mucus renewal and degradation exists, mucin utilization can provide substrate exchange, microbial cross-feeding, and fermentation while preserving mucus barrier integrity. Acetate and propionate are exemplary fermentation products associated with *A. muciniphila*. (**B**) If the rate of community-level mucin degradation exceeds the rate of mucus renewal, mucus thinning may increase microbial access to the epithelium and the risk of inflammation or infection. The illustrated contexts and consequences are exemplar rather than inevitable results of *A. muciniphila* colonization. Large solid downward arrows illustrate mucin degradation; small orange circles illustrate released mucin-derived carbohydrate substrates; dashed red arrows illustrate possible microbial encroachment. Rods represent bacteria, while circular microbiota insets represent microbial community composition. Check marks illustrate favorable consequences and crosses illustrate possible adverse consequences, not experimental measurements. Food illustrations represent dietary contexts rather than direct effects of particular foods. SCFA, short-chain fatty acid. Mucus thickness, bacterial abundance, and molecular structures are not to scale.

Although *A. muciniphila* is widely regarded as a beneficial mucin-degrading commensal, accumulating evidence indicates that its mucinolytic activity can become detrimental under specific ecological and dietary conditions [16]. When mucus degradation becomes excessive or dysregulated, it can erode the protective mucus layer, leaving intestinal cells vulnerable to harmful substances such as bacterial toxins, inflammatory compounds, and pathogens, predisposing the host to colitis and infection. The study revealed that a low-fiber diet increased *A. muciniphila* levels, reduced the thickness of the inner mucus layer, and rendered animals more susceptible to *Citrobacter rodentium*, a pathogen that mimics human *Escherichia coli* infection [76]. This paradox reflects a fundamental concept: mucin degradation is beneficial only when tightly balanced with mucus renewal and microbial ecosystem stability (Figure 6).

For example, in murine models, antibiotic administration followed by oral supplementation with *A. muciniphila* resulted in an “over-colonized” state, leading to a significant reduction in mucus levels and increased inflammatory responses [11]. Other research revealed that when animals were fed a low-fiber diet, *A. muciniphila* levels were elevated and correlated with a diminished mucus layer, increased allergic sensitivity to food allergens, and increased colon inflammation [77]. An additional investigation found that *A. muciniphila* specifically increased host susceptibility to *C. rodentium* infection during fiber deprivation, as its removal from the synthetic community was sufficient to prevent a serious infection. Importantly, when the mice were put back on a fiber-rich diet, the presence of *A. muciniphila* lowered the pathogen burden, demonstrating the mucin specialist’s context-dependent benefits [78].

In addition to fiber, research has shown that glucose concentration influences *Akkermansia* levels in vivo. According to a recent study, mice given drinking water with 10% glucose or fructose showed higher levels of *A. muciniphila* than animals given vehicle control. Analysis of the colonic mucus layer showed that, compared to control animals, gut bacteria were located closer to the epithelial layer, and the inner mucin layer was thinner in glucose-treated mice. When these mice were administered dextran sulfate sodium, they not only had a reduced mucus layer but also developed severe colitis [79].

Changes in *Akkermansia* levels have also been reported in the context of viral infections. Notably, in neonatal mouse pups, rotavirus infection led to an early increase in *Akkermansia* abundance, a phenomenon associated with a reduction in mucus-filled Goblet cells [80]. As discussed in the following reviews, rotaviruses bind to intestinal enterocytes through certain glycans [81]. In silico analysis has demonstrated that the VP8* domain of rotavirus binds mucin glycans containing sialic acid and galactose residues [80]. Interestingly, the addition of intestinal mucin was found to inhibit rotavirus replication [80,82]. Furthermore, it was revealed that mucus degraded by *A. muciniphila* had reduced capacity to suppress rotavirus infection, indicating that mucin may act as a decoy receptor that interferes with rotavirus cell invasion [80]. 

*Akkermansia* levels are similarly elevated in COVID-19 patients [83], and oral administration with live *A. muciniphila* to mice infected with the H7N9 influenza virus attenuated lung damage but did not affect survival rates or weight loss [84]. In addition, *Akkermansia* levels are elevated in coronavirus infections, such as COVID-19; however, whether this increase is ultimately beneficial or detrimental to the host remains to be established [85]. Interestingly, coronaviruses are known to attach to sialic acids, and one study demonstrated they bind to MUC2 from porcine intestinal organoids. The infectivity of transmissible gastroenteritis coronavirus and porcine epidemic diarrhea virus was reduced by the addition of organoid mucus [86]. Therefore, it is possible that excessive mucin degradation by *A. muciniphila* could render the intestinal barrier more permeable to pathogen passage.

## 7. Conclusions

The primary novelty of this work lies in its mechanistic resolution of the “Mucin Paradox”. While recent reviews provide valuable overviews of clinical outcomes or general barrier integrity, they often lack a granular, enzyme-specific resolution. By integrating the latest structural and biochemical data on carbohydrate-active enzymes [20] with the underappreciated roles of mucin-specific proteases and preparatory desulfation or deacetylation steps, this work provides a complete topology of mucin degradation. This review uniquely frames these molecular events within a context-dependent metabolic paradigm. It strongly suggests that the probiotic efficacy of *A. muciniphila* is not an absolute trait. Rather, it is a conditional outcome governed by the precise spatial and temporal regulation of its enzymatic arsenal. Recognizing this nuance is essential for designing next-generation, targeted microbiome therapeutics.

This narrative review positions the bacterium *A. muciniphila* as a functional specialist colonizing the gut niche, and the therapeutic potential of such symbionts is critically dependent on the tightly regulated degradation of the host glycan layer, termed mucin. Therapeutic manipulation of *A. muciniphila* requires acknowledging that its mucin-degrading capacity is inherently double-edged. Barrier integrity is preserved only when enzymatic activity remains synchronized with host mucus production and nutritional availability. While neither inherently beneficial nor detrimental, the probiotic properties of *Akkermansia* have been attributed, at least in part, to enzyme-mediated interactions with the intestinal mucus layer, resulting in a homeostatically balanced mucin degradation. However, benefits may also involve mechanisms beyond active mucin degradation, including bacterial surface proteins such as Amuc_1100 and pasteurized (non-viable) preparations, as reported in Ref. [58]. In fact, this tightly regulated degradation may limit probiotic benefit, potentially even inducing adverse effects in the host. In recent years, the consumption of different probiotic strains has been widely promoted to improve overall health. However, in specific experimental contexts, namely fiber-deprived gnotobiotic or synthetic-community mouse models [76,77,78] and antibiotic-treated mice over-colonized with *A. muciniphila* [11], low fiber availability or over-colonization was associated with increased mucin degradation, a thinner mucus layer, and greater susceptibility to infection or inflammation. These findings are model-dependent; they do not establish that insufficient fiber intake necessarily causes harmful mucus degradation by *A. muciniphila* in humans.

Therefore, from a translational perspective, therapeutic manipulation of these delicate host-microbe relationships through *Akkermansia*-based probiotics, prebiotics, postbiotics, and MDEs may hold considerable promise in developing proactive interventions targeting metabolic and inflammatory disorders. Specifically, in delineating context-specific markers, titers, and culture conditions that maintain a tightly regulated degradation of the host mucus layer for disease management and therapeutic interventions involving this bacterium as a probiotic or postbiotic.

## Figures and Tables

**Table 1:** Key studies summarizing and elucidating the functionalities of *A. muciniphila*.

Experimental Model	Key Findings	Ref.
*In vivo*/*In vitro*	Elucidated regulatory mechanisms of crosstalk between *A. muciniphila*, the host, and other GM members, including effects on colonization, lipid metabolism, immune signaling, and microbial cross-feeding.	[9,25,34,36,37]
*In vitro* (Caco-2, HT-29)	Demonstrated strong adhesion of *A. muciniphila* to human colonic epithelial cell lines and host mucosa, mediated by O-glycan–specific interactions.	[15,17,21]
*In vitro* Gut Reactors	Demonstrated growth of *A. muciniphila* with mucin as the sole carbon source and characterized fermentation outputs, principally acetate and propionate. Butyrate production was reported for the particular isolate and advanced bionic intestinal reactor conditions of Ref. [23] and should not be generalized to the species; in mixed communities, butyrate is attributed to cross-feeding partners.	[18,24,25]
Biochemical Analysis	Characterized an extensive enzymatic arsenal, including 54 glycoside hydrolases and 11 sulfatases acting synergistically to degrade to completion the mucin O-glycans tested.	[20,26,32,38]
Metabolomics/Lipidomics	Revealed synchronized metabolic adaptations—alterations in lipid composition, central carbon metabolism, and energy homeostasis—in response to high mucin availability.	[33,36,39]
Transcriptomics	Confirmed strong upregulation of MDE genes and associated metabolic pathways specifically induced by mucin substrates and mucin-rich environments.	[18,33,40]

**Table 2:** Summary table for key mucin-degrading enzymes of *A. muciniphila* and their functions.

Functional Phase	Enzyme Class	Representative Protein/Gene	Specific Mechanistic Role	Ref.
1. Initial Substrate Preparation	Sialidases (GH33	AmGH33A (Amuc_0625) AmGH33B (Amuc_1835)	Cleaves terminal sialic acid caps to expose underlying glycan structures for downstream processing.	[20,26]
	Fucosidases (GH29, GH95)	AmGH29A (Amuc_0010) AmGH95B (Amuc_1120)	Strips terminal fucose residues, facilitating microbial access to core oligosaccharides.	[20,26]
	Sulfatases	Amuc_0491 Amuc_1655 Amuc_1182	Hydrolyzes sulfate modifications, neutralizing negative charges to permit subsequent glycosidase activity.	[20,35]
2. Glycan Backbone Degradation	Endo-O-Glycanases (GH16)	GH16 family members	Executes internal cleavage of O-glycan chains, accelerating complete mucin depolymerization.	[20,31]
	β-Galactosidases	Amuc_0824 Amuc_1666 Amuc_0771	Hydrolyzes specific galactosidic bonds within mucin carbohydrate side chains during stepwise degradation.	[20,32]
	β-N-Acetylhexosaminidase	Am0868	Degrades N-acetylglucosamine (GlcNAc) and N-acetylgalactosamine (GalNAc) residues released from O-glycans.	[18,20]
3. Protein Scaffold Degradation	Aspartic Protease	Amuc_1434	Cleaves the N-terminal domain of the MUC2 core protein, disrupting disulfide-mediated polymerization.	[9,34]
	Metalloprotease	Amuc_0627 A(AM0627)	Selectively cleaves peptide regions adjacent to O-glycosylated residues within the mucin matrix.	[9,20]
	O-Glycopeptidase	OgpA	Hydrolyzes peptide bonds N-terminal to O-glycan-substituted serine or threonine residues.	[35,38]

## Data Availability

No new data were created or analyzed in this study. Data sharing is not applicable to this article.
